# Synthesis of Modified Nano-Hydrotalcite Clay by Micellar Method and Its Application as Gel-like Crude Oil Flow Improver

**DOI:** 10.3390/gels10070443

**Published:** 2024-07-04

**Authors:** Yingna Du, Michal Slaný, Tianbao Hu, Yubo Lian, Yingxue Bai, Congyu Ke, Gang Chen

**Affiliations:** 1 Shaanxi Province Key Laboratory of Environmental Pollution Control and Reservoir Protection Technology of Oilfields, Xi’an Shiyou University, Xi’an 710065, China; 2 Engineering Research Center of Oil and Gas Field Chemistry, Universities of Shaanxi Provence, Xi’an Shiyou University, Xi’an 710065, China; 3 Department of Materials Engineering and Physics, Faculty of Civil Engineering, Slovak University of Technology, Radlinského 11, 810 05 Bratislava, Slovakia; 4 Department of Materials Engineering and Chemistry, Faculty of Civil Engineering, Czech Technical University in Prague, Thákurova 7, 166 29 Prague 6, Czech Republic; 5 No. 11 Oil Production Plant, PetroChina Changqing Oilfield Company, Xi’an 710021, China; 6 Xi’an Changqing Chemical Group Co., Ltd., PetroChina Changqing Oilfield Company, Xi’an 710068, China

**Keywords:** nano hydrotalcite clay, cationic surfactant, gel properties of crude oil, viscosity, pour point

## Abstract

The network formed by wax precipitation at low temperature and colloid asphaltene at high temperature leads to poor fluidity of heavy oil, and the gelling characteristics of crude oil lead to pipeline blockage, which affects the exploitation, transportation and refining of crude oil. This work prepares a series of cationic surfactant-modified nano hydrotalcite (CSNH) to weaken the network structure and enhance the fluidity of the crude oil by the interaction of organic and inorganic functional groups on the CSNH surface and the components of the crude oil. The results show that CSNHs can all reduce the viscosity of crude oil from different oilfields, among which BTNH can reduce the viscosity of Yanglou (YL) crude oil by 98.8% (31 °C) and depress the pour point by 16.0 °C at most. In the investigation of the universality of crude oil, the modified hydrotalcite was applied to the mixed crude oil (CQH) of Changqing Oilfield, the crude oil (J76) of Jidong Oilfield, the high pour point oil (GN) of Huabei Oilfield, and the crude oil (HQ) of Tuha Oilfield. The viscosity reduction rates were 53.2%, 86.2%, 42.7%, and 63.8%, respectively. The characterization of this nano material confirms the modification of quaternary ammonium cationic surfactant on the surface, resulting in a smaller particle size, and the nano particles are stable under conventional conditions. The mechanism of viscosity and pour point reduction in crude oil by BTNH was discussed by DSC and optical microscopy analysis. The OH- and long-chain alkyl groups on the BTNH surface may interact with the resins, asphaltene and wax through hydrogen bonding and co-crystal, weakening or dispersing their aggregates, thereby improving the fluidity of crude oil. Finally, a cost evaluation was conducted on BTNH, providing useful support for subsequent promotion and application.

## 1. Introduction

The increasing demand for oil has led to massive exploration, which has led to a rapid decline in oil reserves [1,2,3,4], especially for light crude oil. Although some new energy development and utilization is increasing, it is not perfect [5]. Oil resources have proven reserves of about four billion tons distributed in concentrated victory, Daqing, Henan, Liaohe, Xinjiang, the total reserves of more than thirty billion tons, of which the vast majority of the proportion of heavy oil [6,7]. In the process of exploitation, the density and viscosity of heavy oil are greater than that of light crude oil [8,9] because its composition is more complex, containing more wax, gels, resin and asphaltene, mining and transportation are more difficult; and the fluidity is poor at room temperature, and the development cost is very expensive [10,11,12,13]. In order to reduce the viscosity of heavy oil, the problem has become more and more difficult and popular in recent years. At present, people have invested a lot of energy in crude oil viscosity reduction technology [14,15,16]. Heating viscosity reduction, blending light oil viscosity reduction, chemical viscosity reduction, microbial viscosity reduction and ultrasonic viscosity reduction are common technical methods to reduce the viscosity of heavy oil [17,18,19,20]. The methods of viscosity reduction mainly include emulsification, catalytic aquathermolysis, oil-soluble viscosity reducer viscosity reduction, nanomaterial reducer viscosity reduction and so on. Zhang et al. [21] prepared three kinds of viscosity reducers using anionic sulfonates, non-ionic (polyethers and oxyamines) and amphoteric betaine gel and studied the viscosity reduction rate, interfacial tension and emulsifying properties after acting on Daqing crude oil. The results show that erucic acid amide oxide and erucic acid betaine had good viscosity reduction and emulsification effects on Daqing crude oil. The highest viscosity reduction rate can reach 82.24%. Ma et al. [22] synthesized a series of bentonite-supported transition metal complexes for aquathermolysis of heavy oil. On the surface of the experimental results, the viscosity of heavy oil can be reduced by 84.5% by reacting 0.5% catalyst with 10% ethanol at 250 °C for 4 h. Xu et al. [23] used nano-graphene to prepare an oil-soluble viscosity reducer and used the functional groups in the monomer to destroy asphaltene aggregates, thereby improving the fluidity of crude oil. The experimental results show that the viscosity reduction rate reaches 80.0%. Mao et al. [24] explored an effective and inexpensive method for the extraction and transportation of high viscosity and high pour point crude oil, using modified nano-silica particles to reduce the pour point and viscosity of crude oil. The nanocomposites can reduce the viscosity of different heavy oils by more than 60% and depress the pour point by more than 10 °C. Omar F. Al-Mishaal et al. [25] studied the comparison of heavy oil-water thermal upgrading in the presence and absence of molybdenum-based oil-soluble catalysts, and pointed out its potential application in heavy oil in situ upgrading technology. Arash Tajik et al. [26] studied the synthesis of a new ligand for the preparation of Fe, Ni, Co and Cu-based oil-soluble catalysts using sunflower seed oil as an environmentally friendly and inexpensive source. The interaction between metal and ligand is studied by calculation. The experimental results show that the selection of ligands and associated metals is very important to realize the high activity and intelligent catalytic system of the enhanced oil recovery method. At present, there are many other oil production technologies, such as acidizing and optimization by RSM. For example, in the study of Azizollah Khormali et al. [27], acidification was studied in detail, and 2-mercaptobenzimidazole (2-MBI) was used to evaluate the corrosion inhibition of carbon steel in 1 mol HCl solution through experiments and modeling methods.

This paper presents the preparation of modified hydrotalcite using the micelle template method [28,29]. Hydrotalcite, a metal composite hydroxide with an anionic intercalation structure, can be easily synthesized into nano-sized particles [30,31]. With a large specific surface area, it exhibits both hydrophilic and lipophilic properties. By utilizing anionic surfactants with long alkyl chains to modify hydrotalcite, the viscosity can be reduced by 96%, and the pour point can be lowered by up to 14 °C due to its cationic structure and high anion exchange capacity [32]. Drawing from the previous explanation, we hypothesize that nano-hydrotalcite can be effectively modified using cationic surfactants with long-chain alkyl groups. This modification process involves the formation of ionic bonds with anions and cations within the lamellar structure of hydrotalcite. Importantly, this method preserves the -OH functional group in the hydrotalcite structure while ensuring strong, unsaturated, non-directional ionic bonding that adheres firmly to the surface of the hydrotalcite [33]. Hence, the integration of this modified hydrotalcite into crude oil through hydrogen bonding of hydroxyl and long-chain alkyl groups with potent polar functionalities, such as hydroxyl, carboxyl, amino, and mercaptan, present on colloids and asphaltenes, is feasible. This interaction modifies the aggregation pattern of colloids and asphaltenes, potentially influencing their fluid properties. During colder temperatures, crude oil tends to precipitate wax or gel crystals, forming a complex three-dimensional network. The alkyl groups located on the surface of the modified hydrotalcite demonstrate either eutectic properties or adsorption capabilities with the wax crystals, which significantly curtails their expansion and boosts the crude oil’s fluidity in colder temperatures. Therefore, our work mainly uses a series of cationic surfactants with different alkyl side chains as modifiers to evaluate the performance of modified hydrotalcite as a crude oil fluidity improver. At the same time, we also believe that the modified nano hydrotalcite also has the ability to reduce the wax precipitation point. Finally, the mechanism of modified hydrotalcite on crude oil fluidity was speculated by various characterization methods. 

## 2. Results and Discussion

### 2.1. Material Screening and Condition Optimization

**Figure 1:**
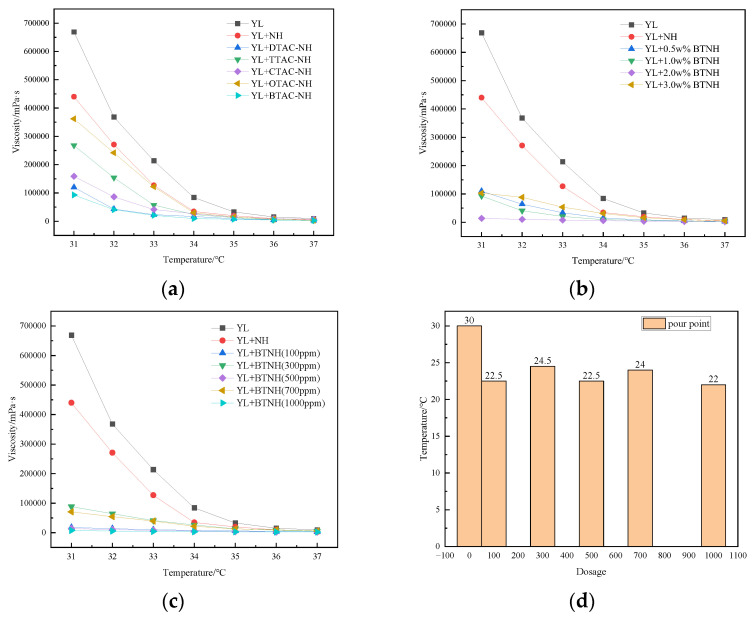
Preparation and optimization of usage parameters (**a**) YL + NH + surfactants, (**b**) YL + NH + various concentrations of BTNH, (**c**) YL + NH + various ppm of BTNH, (**d**) pour point.

A series of quaternary ammonium salt surfactants with different chain lengths were used as modifiers to prepare modified hydrotalcite, and then n-octanol was used as a solvent to prepare suspension for subsequent experimental research. The amount of modified hydrotalcite was controlled to be 500 ppm, and the amount of modifier was 1.0 w %. The effect of modifiers on the viscosity of gel-like crude oil was investigated. It can be seen from Figure 1a that a series of modified hydrotalcites have a certain viscosity reduction effect on YL crude oil. Among the various formulations, the docosyl trimethyl ammonium chloride (BTAC)-modified nano-hydrotalcite (BTNH) displays a particularly pronounced viscosity reduction effect. In comparison to the untreated oil sample, the viscosity of YL oil decreased by a remarkable 86.1% at 31 °C. DTAC-NH follows closely, achieving a viscosity reduction rate of 82.1%. At a fixed concentration of 500 ppm for the modified hydrotalcite, we examined the impact of BTAC dosage on crude oil viscosity. As evident from Figure 1b, a dosage of 2.0 wt% yielded the most significant viscosity reduction, achieving a rate of 97.9%. Having identified these optimal conditions, we further investigated the effect of the modified hydrotalcite quantity on crude oil viscosity. Figure 1d illustrates the pour point of crude oil at varying doses of BTNH. Specifically, Figure 1c highlights that when the modified hydrotalcite concentration was set to 1000 ppm, the viscosity of YL heavy oil reduced by 98.8%, while its pour point decreased from 30.0 °C to 22.0 °C, representing a notable reduction of 8.0 °C.

### 2.2. Universal Applicability Evaluation

**Figure 2:**
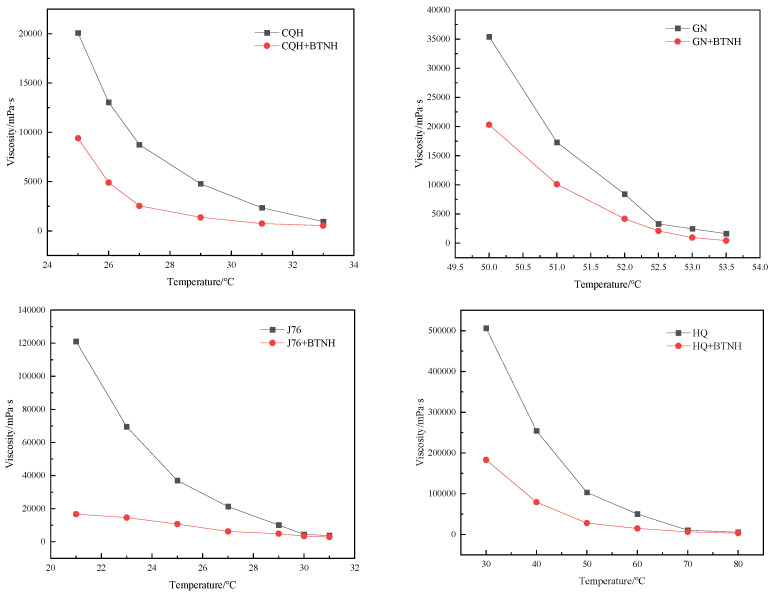
Universal applicability evaluation on different crude oil.

At present, most of the viscosity reducers have specific applicability and lack wide applicability. The structural characteristics of BTNH prepared in this study make it not only prevent wax crystals in crude oil from forming a three-dimensional network structure in the low temperature region but also effectively control the aggregation of resins and asphaltenes in the high temperature range so that the viscosity reduction effect can be achieved at different temperatures. In this study, we examined the effectiveness of BTNH in various crude oil samples originating from diverse sources, namely Changqing Oilfield’s mixed crude oil (CQH), Jidong Oilfield’s crude oil (J76), Huabei Oilfield’s high pour point oil (GN), and Tuha Oilfield’s shallow crude oil (HQ). As depicted in Figure 2 and Table 1, the viscosity reduction rate for BTNH in Changqing Oilfield’s mixed crude oil (CQH) stands at 53.2% at 25 °C, with a pour point depression of 13.5 °C. For Huabei Oilfield’s high pour point oil (GN), the viscosity reduction rate achieved by BTNH is 42.7% at 50 °C, resulting in a pour point reduction from 49.0 °C to 41.0 °C. Notably, in the relatively high temperature range, this reduction rate surpasses 73.2% at 53.5 °C. Jidong Oilfield’s crude oil (J76) exhibited a viscosity reduction rate of 86.2% at 21 °C, coupled with a pour point depression of 16.0 °C. Meanwhile, BTNH’s viscosity reduction rate for Tuha Oilfield’s Haqian crude oil (HQ) stands at 63.8% at 30 °C, accompanied by a pour point depression of 9.5 °C. These findings indicate that the viscosity reducer possesses a certain degree of universality, thus promising broad application prospects.

### 2.3. Differential Scanning Calorimetry Analysis

**Figure 3:**
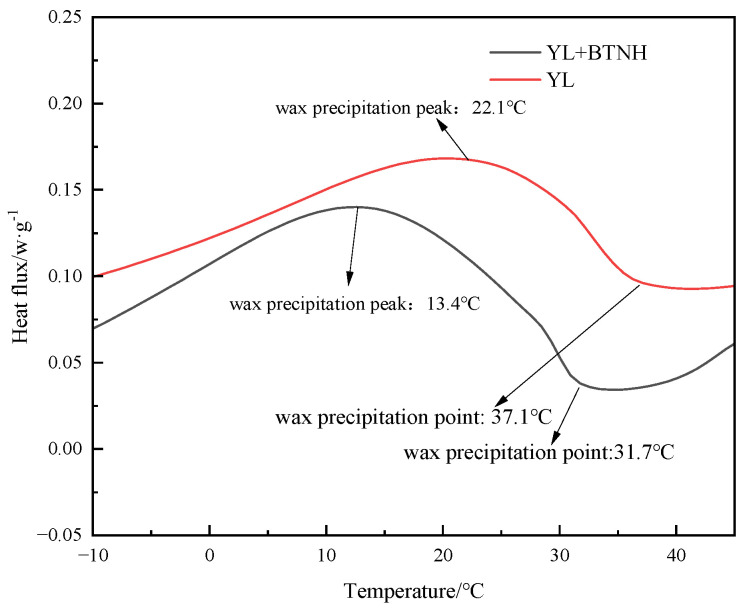
DSC of oil samples before and without and with BTNH.

The differential scanning calorimeter (DSC) was used to analyze the thermal behavior of wax precipitation in crude oil with and without the addition of BTNH. The results, depicted in Figure 3, indicate a decrease in the wax precipitation point from 37.1 °C to 31.7 °C and a reduction in the peak temperature of wax precipitation from 22.1 °C to 13.4 °C. These findings demonstrate the inhibitory effect of BTNH on wax crystal formation during low-temperature wax precipitation, resulting in a lower wax precipitation point. These results align with the impact of BTNH on the viscosity and pour point of crude oil (Table 2) at low temperatures, indicating the effective combination and dispersion of BTNH with wax and gel crystals.

### 2.4. Micro Wax Crystal Morphology Analysis

**Figure 4:**
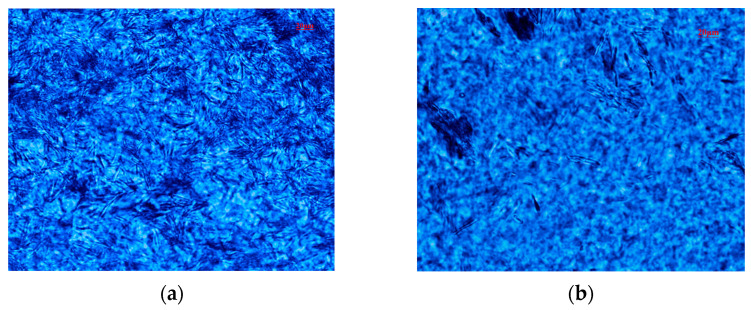
Microscopy of wax in saturated hydrocarbons without (**a**) and with BTNH (**b**).

Using polarizing microscopy, we delved into the impact of BTNH on the crystallization of wax in crude oil’s saturated hydrocarbons. Figure 4a reveals that the wax crystals in untreated saturated hydrocarbons assume a needle-like shape, with a conspicuously aggregated network structure. This aggregation of the three-dimensional network of wax crystals hinders the flow of liquid hydrocarbons, ultimately leading to an increase in viscosity and, ultimately, the loss of fluidity. However, upon the addition of BTNH, as depicted in Figure 4b, the wax crystals still exhibit a needle-like morphology, but there is a marked reduction in their number, and no dense network structure is formed. This indicates minimal impact on the fluidity of liquid hydrocarbons. This observation concurs with the DSC analysis results, thereby directly confirming the capability of BTNH to bind to and inhibit wax and gel crystals, leading to the formation of smaller and more evenly distributed crystal structures.

### 2.5. FTIR Analysis

**Figure 5:**
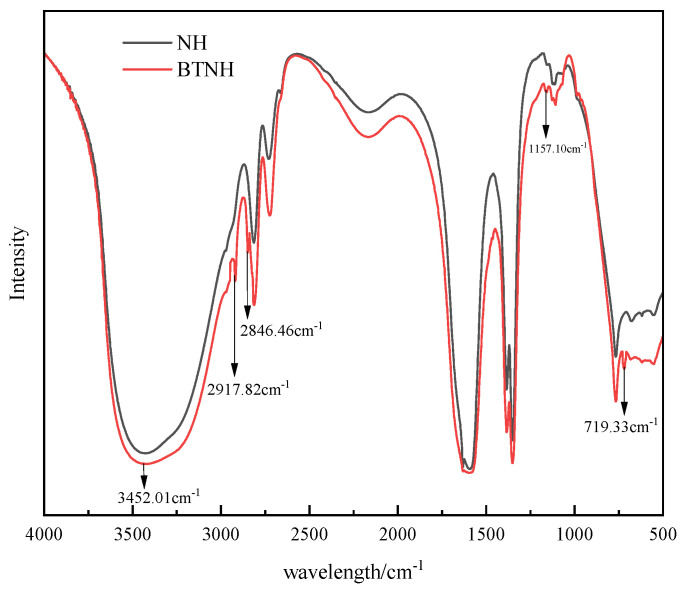
Fourier transform infrared spectroscopy of modified hydrotalcite.

Layered double hydrotalcite (NH) and modified hydrotalcite (BTNH) were analyzed by FTIR, and the results are shown in Figure 5. There was an -CH_3_ stretching vibration band at 2917.82 cm^−1^, and a band at 2846.46 cm^−1^ due to the stretching vibration of -CH_2_. After modification, the new absorption band at 1157.10 cm^−1^ is attributed to the C-N stretching vibration, and the band at 719.33 cm^−1^ is attributed to the -CH_2_ in the plane rocking vibration, which belongs to the weak absorption band. Through the above data analysis, it can be seen that BTAC has been adsorbed on hydrotalcite by ion exchange, forming a novel material of modified hydrotalcite. The broad band of hydrotalcite at 3452.01 cm^−1^ is due to the presence of -OH hydroxyl group. The hydroxyl group will form hydrogen bonds with wax, gels resin and asphaltene in crude oil, hindering the formation of these structures, so as to achieve the effect of viscosity reduction.

### 2.6. Thermogravimetric Analysis

**Figure 6:**
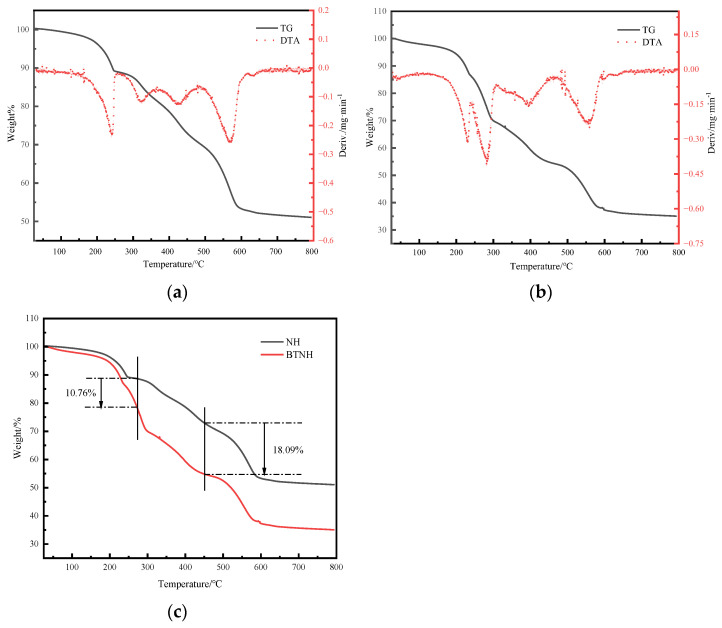
Thermogravimetric of modified hydrotalcite (**a**) TG-DTA curve of NH, (**b**) TG-DTA curve of NH and (**c**) TG curves of NH and BTNH.

The TG-DTA curves in Figure 6a,b show that there are three obvious endothermic peaks on the DTA curve during the thermal degradation of the sample. The endothermic peak at 200–300 °C is due to the loss of water evaporation caused by the removal of adsorbed water on the surface of the hydrotalcite and the loss of crystal water between the laminates [34]. In the range of 300–500 °C, the hydroxyl groups and interlayer carbonate ions are mainly removed in the form of H_2_O and CO_2_, respectively. In the range of 500–600 °C, the endothermic peak corresponds to the process of residual small molecules falling off. The thermogravimetric analysis presented in Figure 6c demonstrates that before reaching 275 °C, BTNH underwent a mass loss of 10.76% compared to the unmodified hydrotalcite. This discrepancy is attributed to the degradation of long-chain alkyl groups present on the surface of the hydrotalcite, causing a reduction in mass. Between 275 °C and 452 °C, the weight loss can be attributed to the elimination of hydroxyl groups and the dissolution of interlayer carbonates [35,36]. Notably, during this temperature range, BTNH exhibited an additional 18.09% mass loss compared to unmodified hydrotalcite. The further decrease in mass is likely attributed to the decomposition of BTAC, occurring either on the external surface or interstitially within the layers of the hydrotalcite. Furthermore, this finding is corroborated by FTIR characterization, which confirms the presence of BTAC in the modified hydrotalcite [37]. 

### 2.7. Contact Angle Evaluation

**Figure 7:**
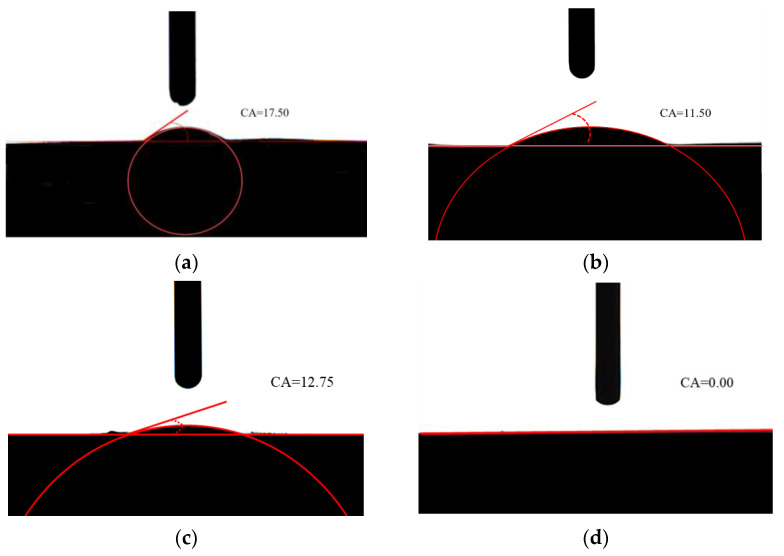
Contact angle of modified hydrotalcite (**a**) NH + distilled water (**b**) NH + oil (**c**) BTNH + distilled water and (**d**) BTNH + oil.

Both the unmodified and BTAC-modified hydrotalcites were individually compressed, and their contact angles were subsequently measured using water and kerosene droplets. The findings are depicted in Figure 7. A comparative analysis of the contact angles reveals that the unmodified hydrotalcite exhibits relatively low angles with both water and oil, indicating its superior hydrophilicity and lipophilicity [38]. Upon modification of hydrotalcite with BTAC, the surface hydrophilicity underwent a notable augmentation, leading to an intensified lipophilicity and a contact angle reaching 0.0°. The increase in lipophilicity signifies an improved dispersion of the modified hydrotalcite in oil, thereby enhancing its affinity and integration with oily compounds to boost effective concentrations.

### 2.8. Dispersivity in Liquid Phase

**Figure 8:**
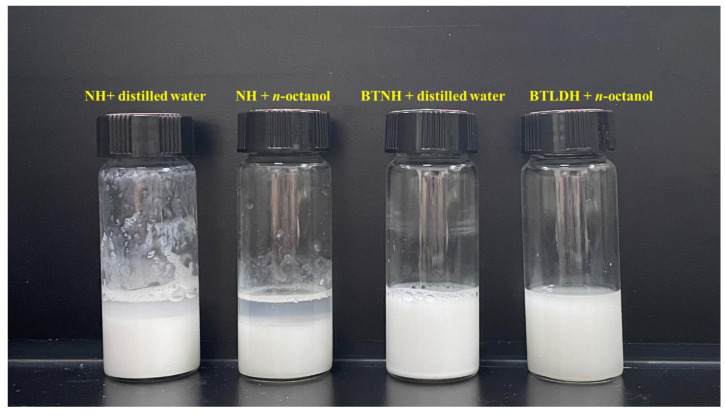
Dispersion and stability of modified hydrotalcites.

In order to further verify the hydrophilic and lipophilic properties of hydrotalcite, the dispersion and stability of modified and unmodified hydrotalcite in distilled water and n-octanol were evaluated. The comparison in Figure 8 shows that the hydrophilicity of the modified and unmodified hydrotalcite is better, but the stability of the unmodified hydrotalcite is poor and easy to settle. Obvious stratification was observed after 1 h. The modified hydrotalcite has better dispersion and stability in n-octanol and is not easy to settle. After 1 h, no stratification has been observed. It can be seen from the above experiments that BTNH can be better dispersed in the organic phase, which is conducive to its role in crude oil.

### 2.9. X-ray Diffraction (XRD) Analysis

**Figure 9:**
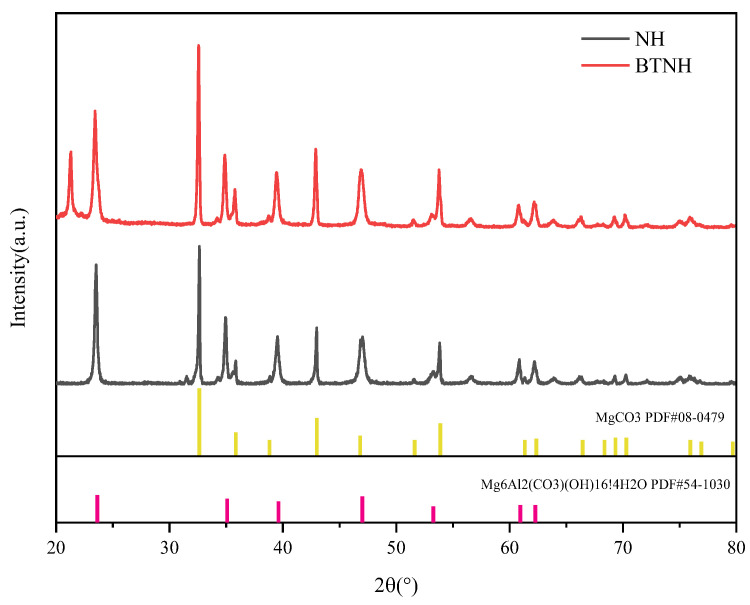
XRD of modified hydrotalcite.

As shown in Figure 9, the XRD of hydrotalcite and BTNH was determined. The (003), (006), (009), (015), (018), (110) and (113) characteristic crystal planes of hydrotalcite appeared at 22.9°, 34.3°, 38.9°, 46.4°, 60.1° and 61.5°, respectively, indicating that the modification did not destroy the crystal structure of hydrotalcite [39,40]. The presence of MgCO_3_ at angles 32.5°, 42.5°, and 53.9° was attributed to the precipitation of carbonate and magnesium ions resulting from urea hydrolysis during the high-temperature hydrothermal synthesis process. Upon comparing the diffraction signal intensities between the two samples, it became evident that the diffraction peak of BTNH exhibited a sharper profile compared to NH, signifying its superior crystallinity.

### 2.10. Scanning Electron Microscope Analysis (SEM) Analysis

**Figure 10:**
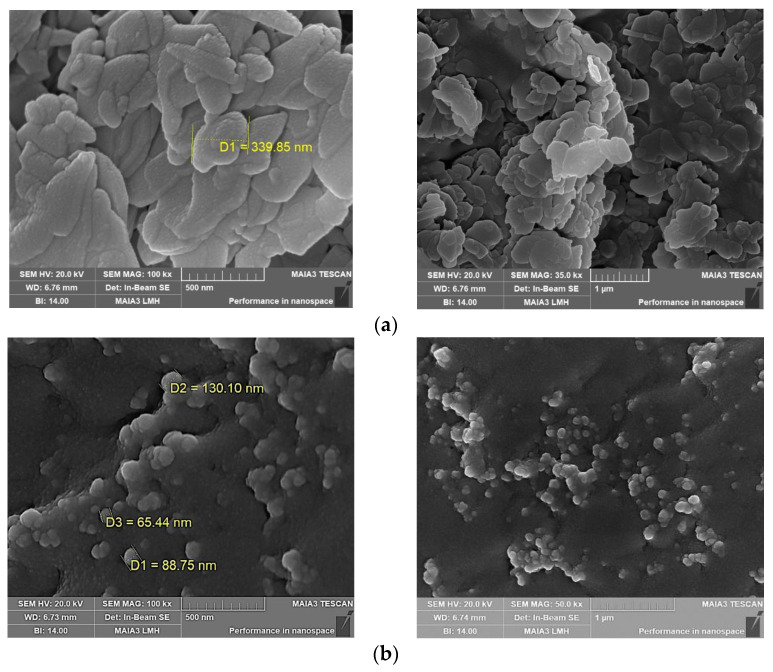
SEM images of modified hydrotalcites with gel morphology (**a**) NH, (**b**) BTNH.

From Figure 10, it can be seen that NH and BTNH have typical lamellar structures, the morphology is basically the same, and the difference is not large. The NHs lamellar structure exhibits a cross-aggregated formation, characterized by a regular and orderly staggered pattern. In contrast, the lamellar structure of BTNH displays a more loosely packed aggregation. The diameter of most lamellar structures of BTNH is smaller than that of NH, and it shows a hexagonal structure. In the conventional state, it can reach nanoscale particles, which can increase the effective concentration of hydrotalcite in crude oil.

### 2.11. Zeta-Potential Particle Size Analysis

**Figure 11:**
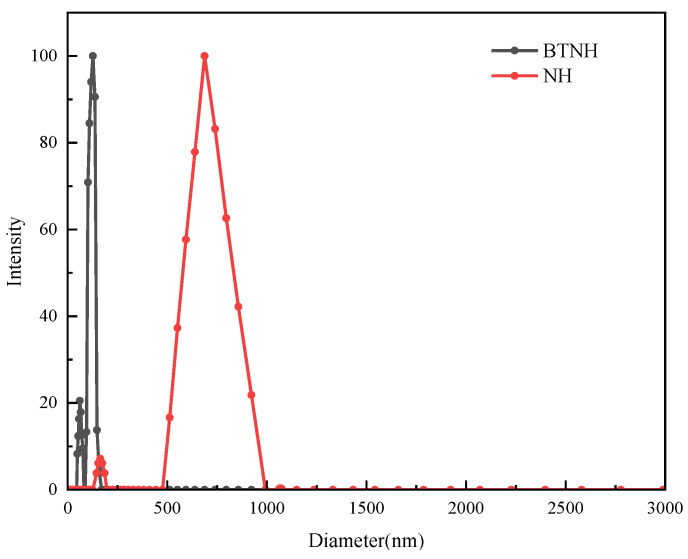
Particle size of modified hydrotalcite.

The results of the scanning electron microscope in the previous section showed that the particle size of the prepared BTNH reached the nanometer level. Therefore, the particle size of the material was characterized by Zeta potential analysis. As shown in Figure 11, the average particle size of BTNH reached 126.8 nm, which was much smaller than that of unmodified hydrotalcite (average particle size of 687.5 nm). According to the experimental results, it can be concluded that BTAC can change the surface properties of the material on the one hand, and on the other hand, it can make the material more evenly dispersed and the particle size smaller.

### 2.12. Mechanism for Improving Crude Oil Fluidity

**Figure 12:**
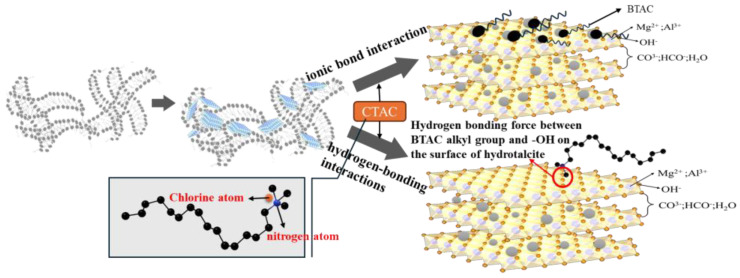
Structure and modification principle of hydrotalcite.

**Figure 13:**
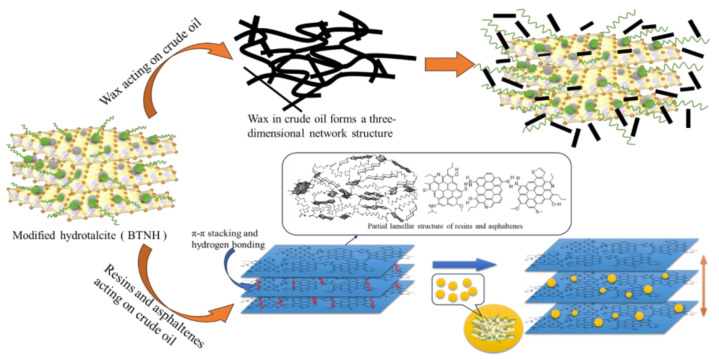
The mechanism of improving crude oil fluidity by BTNH.

Combined with the results of infrared spectroscopy, contact angle experiments and thermogravimetric analysis, it can be explained that the quaternary ammonium salt surfactant adsorbs on the surface of the hydrotalcite to form a surface organically modified hydrotalcite. The modification mechanism is as shown in Figure 12. The quaternary ammonium surfactants form micelles in water, and during hydrothermal synthesis, hydrotalcite is formed on the micellar templates, which results in a smaller particle size of hydrotalcite. The combination of infrared spectroscopy, contact angle experiments, and thermogravimetric analyses can indicate that the quaternary ammonium surfactants adsorb on the surface of hydrotalcite to form the surface-organized modified hydrotalcite, which may adsorb on the surface of particles through the ionic bonding with the anions in the interpolations, and also It may be due to the presence of alkyl side chains in the ammonium surfactant, which makes it produce a hones hydrogen bonding force with -OH on the surface of the hydrotalcite, thus achieving surface modification. Utilizing DSC and polarized light microscopy, it has been observed that BTNH interacts with wax crystals, altering their morphology and inhibiting the growth of wax or gel crystals, ultimately forming a network structure that effectively reduces pour point and viscosity. The quaternary ammonium salt surfactant’s modification of hydrotalcite boosts the dispersion of sheet-like structures, enabling the creation of nanoparticles. Drawing from the aforementioned evaluation and characterization findings, we propose a plausible mechanism for how modified hydrotalcite enhances crude oil flow, as depicted in Figure 13. Resin and asphaltene molecules are the components with the largest molecular weight and the strongest polarity in crude oil. They are complex structures formed by alkyl-branched chains, polycyclic aromatic nuclei and naphthenic aromatic nuclei containing heteroatoms [41,42]. They contain a large number of S, N, O and other heteroatoms, which exist in groups such as thiohydroxyl, amino, hydroxyl and carboxyl groups. Under the action of π-π stacking and hydrogen bonding, these groups are often associated with hydrogen bonding or dipole interaction, resulting in a strong cohesion so that a number of resin and asphaltene molecules aggregate into a layered stacking state. When relative displacement occurs between crude oil molecules, a large internal friction force can be generated, thereby increasing the viscosity of crude oil. Given its alkyl, OH-, and lamellar structures, BTNH engages in hydrogen bonding and van der Waals forces with resins and asphaltenes. This interaction facilitates the dispersion of resin and asphaltene aggregates, thus diminishing the viscosity of these components. During the low-temperature phase, where wax crystals precipitate from crude oil, the modified hydrotalcite’s long-chain alkyl groups undergo eutectic formation or adsorption with the wax crystals, as reported in previous studies [43]. Given its nanoscale particle size and high specific surface area, BTNH exhibits excellent dispersibility for wax and gel crystals, effectively inhibiting their aggregation and promoting the formation of a network structure. Concurrently, the base structure of hydrotalcite can distort the wax crystal’s formation and hinder its expansion. The combined effect of these two mechanisms leads to a reduction in crude oil’s pour point and viscosity at lower temperatures.

### 2.13. Cost Accounting and Feasibility Analysis

The development of this crude oil fluidity enhancer utilized magnesium nitrate, aluminum nitrate, and urea as primary ingredients. Balancing viscosity reduction potential with cost considerations, dodecyl trimethyl ammonium chloride was chosen as the optimal modifier. Furthermore, factors such as energy usage, packaging expenses, transportation costs, and labor charges are taken into account. As a result, the overall cost of DTAC-NH, calculated using Formula (1), is approximately 11,927.9 ¥/ton. However, the current polymer viscosity reducers, such as commercial-grade EVA, are priced at 30,000 ¥/ton, and most surfactants used in crude oil, such as industrial-grade octadecyl dimethyl ammonium chloride, are priced at 17,000 ¥/ton. Therefore, the DTAC-NH crude oil flow improver has an obvious commercial competitive advantage and has a very broad application prospect.

(1) $$C=M_{0}\cdot C_{0}+M_{1}\cdot C_{1}+M_{2}\cdot C_{2}+M_{3}\cdot C_{3}+C_{X}+C_{Y}$$

where C is the total cost of the DTAC-NH crude oil flow improver, ¥/ton; M_0_ is the proportion of urea in the flow improver of crude oil containing DTAC-NH per ton, which is 0.3196 tons/ton; C_0_ is the cost per ton of urea, 1700 ¥/ton; M_1_ is the proportion of magnesium nitrate in the flow improver of crude oil containing DTAC-NH per ton, which is 0.4095 tons/ton; C_1_ is the cost of magnesium nitrate per ton, 1900 ¥/ton; M_2_ is the proportion of aluminum nitrate in the flow improver of crude oil containing DTAC-NH per ton, which is 0.1997 tons/ton; C_2_ is the cost of per ton of aluminum nitrate, 3800 ¥/ton; M_3_ is the proportion of DTAC in each ton of crude oil flow improver containing DTAC-NH, 0.0712 tons/ton; C_3_ is the cost of DTAC per ton, 13,000 ¥/ton; C*_Y_* is the labor cost and packaging cost per ton of DTAC-NH production, ¥/ton.

(2) $$C_{X}=Q\cdot C_{e}$$

where C_e_ is the price of China’s industrial electricity, taking 0.725 ¥/kW·h; Q is the power consumption of producing 1 ton DTAC-NH, kW·h; C_X_ is the total cost of energy consumption per ton of DTAC-NH production, ¥/ton.

## 3. Conclusions

This study focused on the synthesis of a series of cationic surfactant-modified nano hydrotalcite (CSNH) compounds to enhance crude oil flow. Our experimental findings reveal that the hydrotalcite modified with 2.0 w % of BTAC demonstrates the most prominent viscosity-reducing capabilities, achieving a peak reduction rate of 98.8% at 31 °C. Moreover, BTNH has a certain universality, breaking the limitations of traditional crude oil flow improvers and having great application prospects. The changes in crude oil before and after the action of modified hydrotalcite were investigated by DSC and polarized light microscopy. It was found that the wax precipitation point is depressed by 5.4 °C, and the wax and gel crystal morphology changes from the original dense state to the sparse state and forms a needle shape. Subsequently, the properties of this nanomaterial were thoroughly examined using various techniques, including Fourier Transform Infrared Spectroscopy (FTIR), Thermogravimetric Analysis (TGA), Contact Angle Measurement, Scanning Electron Microscopy (SEM), X-ray Diffraction (XRD), and Zeta Potential Particle Size Analysis. Combined with the properties of the materials and the characterization of the oil samples, we proposed the possible mechanism of the modified hydrotalcite on the pour point and viscosity reduction in crude oil. Cost accounting shows that the cost of the BTNH crude oil flow improver developed in this work is lower than that of similar products in the current market and has an obvious commercial competitive advantage. This research work can provide a useful reference for research in the field of crude oil fluidity.

## 4. Materials and Methods

### 4.1. Materials

The crude oils used in this study are Yanglou crude oil (YL) from Henan Oilfield, mixed gathering and transportation crude oil (CQH) from Changqing Oilfield, crude oil (J76) from Jidong Oilfield, high pour point oil (GN) from Huabei Oilfield, and Haqian crude oil (HQ) from Tuha Oilfield. The components of crude oil are determined by the polar separation method. The main components of crude oil are saturated hydrocarbons, aromatic hydrocarbons, asphaltenes and resins. The content of each component in the heavy oil sample (SARA) was analyzed according to the SY/T 5119-2008 standard, and the analysis results are shown in Table 3. The reagents involved in this experiment were all analytically pure and purchased from the Tianjin Damao Chemical Reagent Factory.

### 4.2. Preparation of Modified Nano Hydrotalcite

M_x_N_y_ + zH_2_O = xM^z^ + (aq) + yN^z−^(aq) (3)

**Figure 14:**
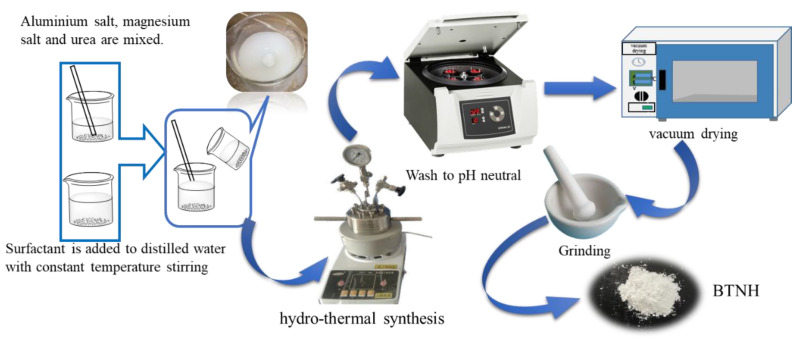
Preparation process of modified hydrotalcite.

Hydrotalcite was prepared by hydrothermal synthesis (Figure 14). The molar ratio of Mg(NO_3_)_2_/Al(NO_3_)_3_ was fixed at 3:1. In an alkaline environment, urea was used to provide alkaline environment in this paper, and the amount of urea was 2.5 times the total molar ratio of Mg/Al. The modified hydrotalcite was prepared by adding a mixed solution of modifier and solvent during the preparation of un-modified nano hydrotalcite (NH). The amount of surfactant was 2.0 wt% of distilled water, and 0.3 wt% salicylic acid was added. After the surfactant and inorganic were dissolved and mixed, they were added to the reactor and reacted at 160 °C for 6 h. After the reaction is completed, cool to room temperature, centrifuge settings: 4000 r/min, once every 10 min centrifugal washing, until pH = 7. Then, it was placed in a vacuum drying oven, dried at 70 °C, and finally obtained the modified hydrotalcite. A certain amount of modified hydrotalcite was added to *n*-octanol, and the ultrasonic time was 30 min to the uniform state. Different concentrations of suspension were prepared for subsequent experiments.

### 4.3. Evaluation of Modified Nano Hydrotalcite in Crude Oil

The pour point of crude oil was determined according to SY/T 2541-2009, and the viscosity was determined according to the SY/T 0520-2008 standard.

### 4.4. FTIR Analysis

Fourier transform infrared spectroscopy was analyzed according to GB/T 6040-2019. The infrared spectra were measured (1 mg of a sample homogenized with 200 mg KBr) using the Nicolet 6700 Fourier Transform Infrared (FTIR) spectrometer from company Thermo Scientific™ (Amsterdam, The Netherlands).

### 4.5. Contact Angle Determination

The contact angle measurement method is GB/T 30447-2013. The analysis method of the test results is the conic curve method or the tangent method [44].

### 4.6. Dispersivity Experiment

The same mass (1 g solid powder, 10 mL solvent in this work) of modified and unmodified hydrotalcite were placed in a special sample bottle, and distilled water and n-octanol were added, respectively. Ultrasonic treatment was performed for 3 h, and the phenomenon was observed after standing at room temperature.

### 4.7. X-ray Diffraction (XRD) Analysis

The test and analysis methods were carried out according to GB/T 37983-2019. The material was measured and analyzed by Brook-D8 ADVANCE X-ray polycrystalline diffractometer.

### 4.8. Scanning Electron Microscope (SEM) Analysis

The microstructure of the material was measured and analyzed by TESCAN (SEM) field emission scanning electron microscope. The test method is implemented according to JY/T 0584-2020. 

### 4.9. Zeta-Potential Particle Size Analysis

During this experiment, the particle size analysis was conducted in adherence to the GB/T 32668-2016 standard. Specifically, the Brookhaven-NanoBrook Omni type of particle size and zeta potential analyzer were utilized to determine the material’s particle dimensions.

### 4.10. Thermogravimetric Analysis (TGA)

The test analysis method was carried out according to GB/T 27761-2011. The TGA was carried out on a Mettler-Toledo TGA/DSC 8220 in Switzerland with a temperature range of 25–800 °C, nitrogen atmosphere and a ramp-up rate of 10 °C/min. The 50  ±  0.1 mg of powder sample was used for analysis.

### 4.11. Differential Scanning Calorimetry (DSC) Analysis

Wax precipitation point test method according to the standard SY/T 0545-2012.

### 4.12. Optical Microscope Analysis

The saturated hydrocarbons were separated from crude oil using SY/T 5119-2016 method. The test analysis method is referenced in the literature [45].

## Figures and Tables

**Table 1:** Drilling fluid evaluation of MMHlc at 25 °C and 250 °C.

Temperature	Recipe	AV/ mPa·s	PV/ mPa·s	YP/ Pa	FL/ mL	Lubricity Factor	YP/PV
25 °C	4% base mud	5.05	4.80	0.25	48.5	0.23	0.05
	4% S1	6.05	2.00	4.05	13.5	0.25	2.03
	4% S2	6.60	2.10	4.50	16.7	0.27	2.14
	4% S3	7.75	3.00	4.75	22.1	0.22	1.58
250 °C	4% base mud	2.75	2.70	0.05	150.0	/	0.02
	4% S1	5.70	2.00	3.70	67.5	0.14	1.85
	4% S2	5.25	1.90	3.35	45.6	0.15	1.76
	4% S3	3.00	1.50	1.50	62.1	0.11	1.00

**Table 2:** Effect of BTNH on the pour point of different crude oil.

Crude Oil	YL	CQH	J76	GN	HQ
Pour point/°C	30.0	24.0	21.0	49.0	15.5
Pour point with	22.0	10.5	5.0	41.0	6.0

**Table 3:** Physical property analysis of crude oil.

Crude Oil	Pour Point/°C	Saturated HC/%	Aromatic HC/%	Asphaltene/%	Resin/%
YL	31.0	45.63	33.24	8.31	12.82
CQH	24.0	52.70	24.60	10.02	9.26
J76	20.5	49.52	31.43	7.23	11.82
GN	49.0	62.45	20.12	10.41	7.02
HQ	15.5	30.05	21.15	30.17	18.63

## Data Availability

The data presented in this study are openly available in article.
